# Growing as slow as a turtle: Unexpected maturational differences in a small, long-lived species

**DOI:** 10.1371/journal.pone.0259978

**Published:** 2021-11-18

**Authors:** Devin Edmonds, Michael J. Dreslik, Jeffrey E. Lovich, Thomas P. Wilson, Carl H. Ernst

**Affiliations:** 1 Illinois Natural History Survey, University of Illinois at Urbana-Champaign, Champaign, Illinois, United States of America; 2 U.S. Geological Survey, Southwest Biological Science Center, Flagstaff, Arizona, United States of America; 3 Department of Biology, Geology and Environmental Science, University of Tennessee, Chattanooga, Tennessee, United States of America; 4 Department of Biology, George Mason University, Fairfax, Virginia, United States of America; Texas A&M University, UNITED STATES

## Abstract

Turtle body size is associated with demographic and other traits like mating success, reproductive output, maturity, and survival. As such, growth analyses are valuable for testing life history theory, demographic modeling, and conservation planning. Two important but unsettled research areas relate to growth after maturity and growth rate variation. If individuals exhibit indeterminate growth after maturity, older adults may have an advantage in fecundity, survival, or both over younger/smaller adults. Similarly, depending on how growth varies, a portion of the population may mature earlier, grow larger, or both. We used 23-years of capture-mark-recapture data to study growth and maturity in the Spotted Turtle (*Clemmys guttata*), a species suffering severe population declines and for which demographic data are needed for development of effective conservation and management strategies. There was strong support for models incorporating sex as a factor, with the interval growth model reparametrized for capture-mark-recapture data producing later mean maturation estimates than the age-based growth model. We found most individuals (94%) continued growing after maturity, but the instantaneous relative annual plastral growth rate was low. We recommend future studies examine the possible contribution of such slow, continued adult growth to fecundity and survival. Even seemingly negligible amounts of annual adult growth can have demographic consequences affecting the population vital rates for long-lived species.

## Introduction

Studies of life-history traits are increasingly recognized for their importance regarding species conservation, especially for turtles and tortoises [1,2]. Body size is positively correlated with sexual maturity, reproductive output, and survival [3], so growth patterns play a key role in determining population demography. For instance, slow growing individuals will have lower lifetime reproductive output if they mature later than fast growers yet have the same lifespan, reducing their contribution to population growth. Similarly, slow growers stay smaller for longer as juveniles and/or may mature at smaller sizes, potentially increasing mortality risk from predation. Freshwater turtles are model organisms for studying growth as they can be individually identified with near-permanent markings, plastral scute growth rings allow for age estimation, and their hard shell is easily measured and does not typically fluctuate in size [4,5]. Yet, due to their long lifespan and delayed maturity, it often takes decades of fieldwork to examine growth patterns; thus, turtle growth remains an area of research with many unanswered questions [6].

An unsettled research area relates to indeterminate growth, which has often been assumed to be the norm for ectothermic vertebrates [7,8]. However, recent studies have shown the situation is more complicated, with snakes, lizards, and crocodilians exhibiting determinate growth patterns [9,10]. The case for turtles is less clear. Congdon et al. [11] found an average of 19% of individuals across nine freshwater turtle species that stopped growing after maturity, with populations consisting of a range between determinate and indeterminate growers. When individuals continue growing as adults, survival and fecundity are expected to increase because of their link to body size [12]. Even seemingly negligible growth after maturity [4] can significantly change vital rates in turtle populations [13]. Consequently, determining what proportion of turtles in a population exhibit indeterminate growth can expand our understanding of variation in life-history patterns and improve demographic models.

Individual growth models have been widely used for freshwater turtles [e.g., 14–17] but often without consideration for which model best reflects the growth trajectory present in data. The commonly used von Bertalanffy equation shows a steady exponential decline in growth rate towards asymptotic size, whereas others like the Gompertz and logistic equations produce sigmoidal growth curves [18]. The various growth models can be compared in an information-theoretic framework to identify which is most parsimonious [19], though such methods have only recently been used [e.g., 20,21]. Also, considering sexual variation in growth is a common feature of many species [7,22–24], sex must also be incorporated into models, otherwise important demographic implications are easily overlooked.

The Spotted Turtle (*Clemmys guttata*) is a small freshwater turtle assessed as Endangered by the IUCN [25]. It is distributed across wetlands in the Great Lakes region and along the Atlantic coast of North America. Over the past several decades, the species has experienced severe population declines [26–28], and demographic models are increasingly being used to assess Spotted Turtle population status and inform conservation [29–32]. Such models require estimates of demographic parameters such as age of maturity. Additionally, reproductive output increases with body size [33]; thus, growth analyses can help identify patterns of fecundity and recruitment. Though several growth studies have focused on Spotted Turtles [5,34,35], questions remain about age of maturity, growth rate variation, and whether determinate or indeterminate growth patterns are typical for the species.

We used a historical dataset from a 23-year capture-mark-recapture study (see [36]) on a Spotted Turtle population in southeast Pennsylvania to examine growth and maturity. Specifically, we sought to identify the population’s overall growth pattern, determine if and how sex affected trajectories, and assess generated growth curves’ estimated ages of sexual maturity given a known minimum size of maturity. If growth varies by sex, size of sexual maturity will be reached at different ages for males and females. We also compared interval and age-specific growth models to determine if they provide comparable results, and in effect, validate or invalidate age estimation from plastral scute growth rings, which can be unreliable due to observer error and turtles developing more than one ring per year [37]. Lastly, we investigated whether growth among the adult portion of the population showed an indeterminate, determinate, or mixed pattern because if turtles continue growing as adults, there could be a fecundity or survival advantage for the oldest individuals when such demographic traits are linked to body size.

## Materials and methods

### Study site and data collection

Data were collected annually from 1965 to 1988 as part of a long-term study in Lancaster County, Pennsylvania [33]. The site consists of 25 acres of privately-owned woodland and marsh surrounding an impoundment of a tributary to the Susquehanna River (see [36] for additional details). Turtles were captured by hand and with dipnets and individually marked following Cagle [38], whereby rectangular notches are cut into marginal scutes and numbered anteriorly to posteriorly. Most fieldwork was conducted March–June (903 of 1,004 total captures) when Spotted Turtles are active and before the wetlands dried during mid-summer. Over 23 years, there were 1,004 captures of 425 individuals, with 178 individuals recaptured at least once. Of the 178 recaptured individuals, 161 had at least 30 days between captures and were used for interval growth models. Upon capture, date, time, sex, age, and morphometrics were recorded. Sex was determined based on the characteristics described in Ernst and Lovich [39], including plastron concavity, chin and eye coloration, tail thickness, and tail length. Straight-line plastron length (PL) was measured to the nearest 0.1 mm with dial calipers. We counted the number of discernible pectoral scute annuli following Sexton [40] to estimate age for age-specific growth modeling. After collecting data, turtles were released at the site of capture. Data collection took place before requiring Institutional Animal Care and Use Committee protocols for field studies. Permits were provided to CHE by the Pennsylvania Fish and Boat Commission.

### Growth model selection

We used an information-theoretic approach to select among the von Bertalanffy, Gompertz, and logistic growth equations reparametrized as interval models [41] with the days between capture occasions (Table 1). Ordinary least squares nonlinear regression analyses were conducted in R version 3.6.2 [42]. We used only the first and last capture of each turtle and removed all capture intervals <30 days, resulting in a dataset of 161 individuals (96 females and 65 males; S1 Table). To determine the best model for the pooled dataset, we followed Dodd and Dreslik [43] by evaluating models with AIC_C_ [44]. The effect of sex was assessed by coding with binary variables. For sex variable 1 (*S*_*m*_), we gave males a "1" and females a "0," whereas for sex variable 2 (*S*_*f*_), we gave males a "0" and females a "1". We then replaced each parameter with its sex-specific component, for example, replacing asymptotic size *A*_*∞*_ with (*S*_*m*_*A*_*m*_ *+ S*_*f*_*A*_*f*_). Thus, when considering only males, the component *S*_*f*_*A*_*f*_ reduces to zero, as does *S*_*m*_*A*_*m*_ when considering only females. We then plotted the most parsimonious model(s) for comparisons of growth up to age 40 and the mean hatchling PL for the population (25.6 mm, *s*_*error*_ = 0.063, *n* = 79).

**Table 1:** Equations for age-specific and interval models of individual growth used in the study. Parameters are *t–*age (in years or days), *PL*_*t*_−size at age *t*, *k*–characteristic growth rate coefficient, *A*_*∞*_*−*asymptotic size, *b*–proportion of growth remaining toward *A*_*∞*_ at *t*_*0*_, *e* is the base of the natural logarithms, *PL*_*C*_ is the size at *t*_*1*_, *PL*_*R*_ is the size at *t*_*2*_, and *Δt* is the time interval (*t*_2_ –*t*_1_).

Model	von Bertalanffy [41,45]	Logistic [46,47]	Gompertz [43,48]
Age-Specific	${PL}_{t}=A_{\infty }(1-be^{-kt})$	${PL}_{t}=\frac{A_{\infty }}{\left(1+be^{-kt}\right)}$	${PL}_{t}=A_{\infty }e^{-{be}^{-kt}}$
Interval	${PL}_{R}=A_{\infty }-(A_{\infty }-{PL}_{C})e^{-k\Delta t}$	${PL}_{R}=\frac{{A_{\infty }PL}_{C}}{\left({PL}_{C}+(A_{\infty }-{PL}_{C})e^{-k\Delta t}\right)}$	${PL}_{R}=A_{\infty }{\left(\frac{{PL}_{C}}{A_{\infty }}\right)}^{e^{-k\Delta t}}$
Age Estimate	$t=-\frac{ln(\frac{A_{\infty }-{PL}_{t}}{A_{\infty }b})}{k}$	t=−ln(−1+(A∞PLt)b)k	t=−ln(−ln(PLtA∞)b)k
Velocity	$A_{\infty }be^{-kt}k$	$\frac{A_{\infty }be^{-kt}k}{{\left(1+be^{-kt}\right)}^{2}}$	${-A}_{\infty }b^{-bk-e^{-bk}t}$

### Predicted ages of sexual maturity

We inferred the ages of sexual maturity from growth models using a minimum size at maturity of 80 mm PL, previously determined from field observations and gametogenic data for the population [33,34,49]. Solving models for age at PL = 80 mm, we calculated the range of ages at maturity predicted by the growth function using the estimated parameters for the best-fit model and their respective 95% confidence intervals. We then assessed how long it took turtles to reach this minimum size threshold. Finally, we compared estimates from the growth models of age at maturity to others reported in the literature for the population at our study site [33,49,50].

### Interval versus age-specific models

We used a Kolmogorov-Smirnov Cumulative Probability Test [51] to determine if the proportional growth toward *A*_*∞*_ differed between interval and age-specific growth models. We also repeated all the above analyses and estimations using the age-specific models for comparison with interval models. We used the age at initial capture of an individual for the age-specific models because using all captures introduces pseudoreplication. The age-specific dataset consisted of 79 males and 132 females. Before running age-specific models, we calibrated the ages of turtles to an estimated date of nest emergence. With growth ring counts, age estimates are assumed to comprise a whole year, meaning a turtle with nine rings would be nine years old but, in fact, could be 8.5–9.5 years old, thus adding variation. We used 1 September as our estimated emergence date. We chose the date because the majority (estimated up to 79%) of Spotted Turtles at the study site emerged from nests between August and October. Using this assumption, a turtle aged at one and captured on 1 October is estimated to be 1.08 ((365+30)/365) years old, and a turtle captured on 1 August is 0.92 ((365–31)/365) years old. We then parameterized the age-specific growth model for sex the same as the best-fit interval model and compared results.

### Indeterminate or determinate growth

To determine if turtles showed indeterminate or determinate growth, we focused on a subset of adults (n = 100). First, we parsed the data, retaining only turtles with a PL at first capture >80 mm and a time between captures >365 days. Negative growth assumed to be measurement error was set to zero, leaving a final dataset of 40 males and 60 females. We then calculated the relative annual instantaneous growth rates using a modification of Brody’s formula [52]:

$$\Delta GR=\frac{ln{PL}_{R}-ln{PL}_{C}}{\left(t_{2}-t_{1}\right)/365}$$

where *ln* is the natural logarithm, *PL*_*C*_ is size at first capture, *PL*_*R*_ is size at last capture, and (*t*_2_–*t*_1_) is time between first and last capture in days. Δ*GR* is the instantaneous relative rate of annual plastral growth. Finally, we created a histogram of the *ΔGR* and calculated the percent of individuals showing growth (indeterminate) and no growth (determinate).

## Results

### Model selection

**Fig 1:**
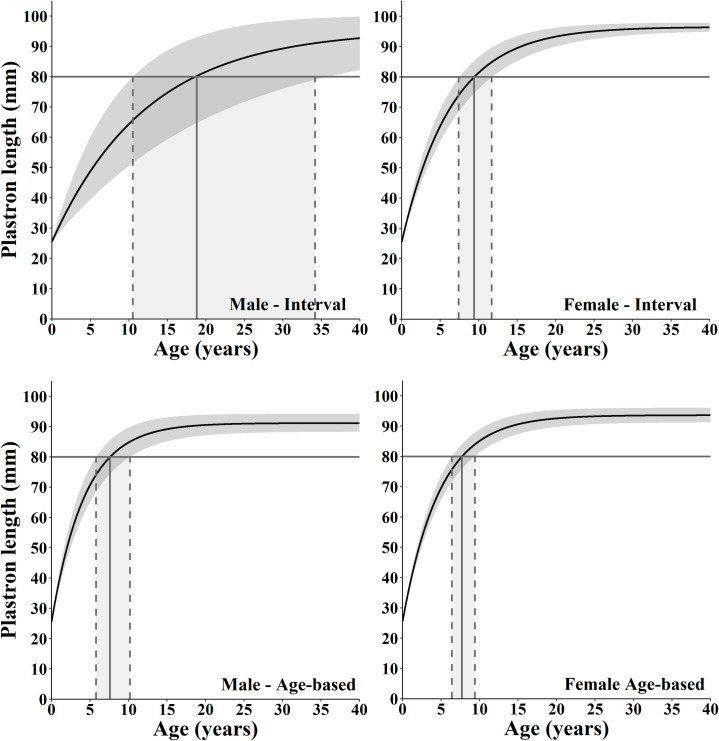
Growth curves for male and female Spotted Turtles (*Clemmys guttata*) from interval and age-based von Bertalanffy growth models. The horizontal lines represent the size of sexual maturity for the population previously determined from field observations and gametogenic data, and the vertical drop lines represent where the specific growth trajectory and its 95% confidence intervals intersect the age of sexual maturity.

**Fig 2:**
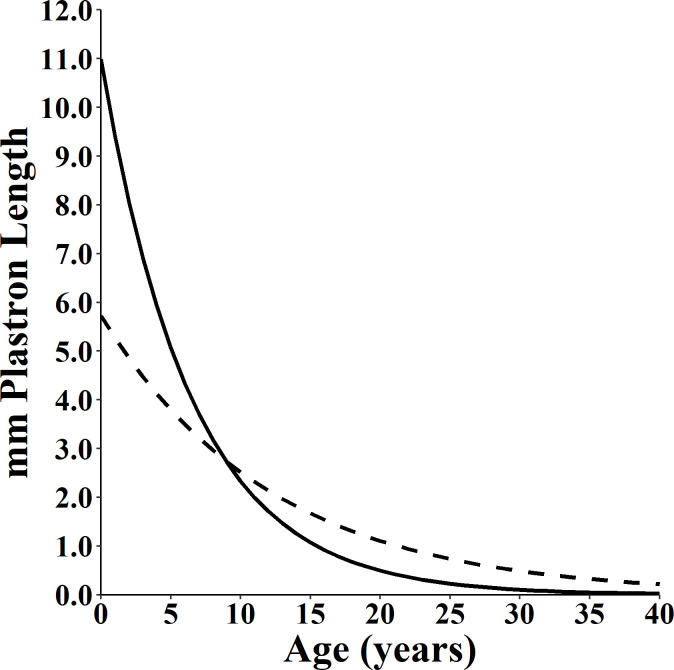
Velocity of Spotted Turtle (*Clemmys guttata*) growth. The figure was generated using the interval von Bertalanffy growth model parameterized to include the effect of sex. Males are the dotted line and females are the solid line.

We found clear support for growth models incorporating sex as a factor, with the sex-based von Bertalanffy best fitting the data (Table 2; Fig 1). The asymptotic size (*A*_*∞*_) estimate of plastron length for males was 95.3 mm (95% C.I. 92.5–99.7 mm) and for females 96.5 mm (95% C.I. 95.3–97.8 mm). The characteristic growth rate (*k*) of males was 0.082 yr^-1^ (95% C.I. 0.048–0.123) and for females 0.155 yr^-1^ (95% C.I. 0.128–0.189). The greater estimate of *k* for females was reflected in a faster growth velocity (Fig 2).

**Table 2:** Adjusted Akaike Information Criterion (AIC_c_) results of nonlinear regression fitting for Spotted Turtle (*Clemmys guttata*) growth equations and their sex-specific counterparts. The candidate models are sorted by ΔAIC_c_ where *K* = number of parameters and *w*_*i*_ = Akaike weight.

Model	*K*	AIC_c_	ΔAIC_c_	*w*_*i*_
von Bertalanffy-Sex	5	799.10	0.00	0.97
Gompertz-Sex	5	806.08	6.98	0.03
Logistic-Sex	5	813.39	14.29	0.00
von Bertalanffy	3	830.16	31.07	0.00
Gompertz	3	839.19	40.09	0.00
Logistic	3	848.34	49.24	0.00

### Ages of sexual maturity

Using the rearrangement of the growth models (Table 1) and the parameter estimated from the best fit model, we produced the range of ages when individuals could reach 80 mm PL (Table 3; Fig 1). On average, the interval model showed males maturing in 18.58 years and females in 9.37 years, and the age-based model showed males maturing in 7.36 years and females in 7.71 years (Table 3). The uncertainty in male growth trajectories from the interval model translated into a broader range of ages of sexual maturity compared to females (Table 3). Of note, the upper confidence interval for males from the interval model (33.87 years) may not be biologically realistic (Table 3; Fig 1).

**Table 3:** Parameter estimates, 95% confidence intervals (C.I.), and age estimates of sexual maturity for the Spotted Turtle (*Clemmys gutatta*) from interval and age-based von Bertalanffy growth models. Parameters are *A*_*∞*_ = asymptotic size in mm, *k* = characteristic growth rate coefficient, Age = age at maturity in years.

Model	*A*_*∞*_	95% C.I.		*k*	95% C.I.		Age	95% C.I.	
Interval Male	95.34	92.51	100.27	0.082	0.050	0.128	18.58	10.90	33.87
Interval Female	96.45	95.27	97.85	0.156	0.130	0.188	9.37	7.37	11.82
Age Male	89.92	87.46	92.55	0.254	0.218	0.297	7.36	5.64	9.70
Age Female	93.63	91.33	96.01	0.209	0.189	0.228	7.71	6.49	9.31

### Interval versus age-specific growth curves

Interval and age-specific growth models depicted the same cumulative probability structure of growth toward their respective *A*_*∞*_ for females (*D*_*max*_ = 0.286, *D*_*crit*_ = 0.294, *p* = 0.365), but not for males (*D*_*max*_ = 0.667, *D*_*crit*_ = 0.294, *p* < 0.001). For both sexes, estimates of *A*_*∞*_ were lower and estimates of *k* were greater for the age-specific model compared to the interval model (Table 3; Fig 1). Additionally, the age-specific model did not capture the largest turtles in the population because we used only the individual’s first age estimated, not subsequent recaptures. Consequently, the confidence intervals for the estimates of *A*_*∞*_ in the age-specific curve do not bound the upper limits of observed values; four males grew larger than 92.55 mm PL and two females grew larger than 96.01 mm PL. Lastly, there was greater certainty in age estimates from the age-specific model when compared to the interval model (Fig 1; Table 3).

### Indeterminate or determinate growth

**Fig 3:**
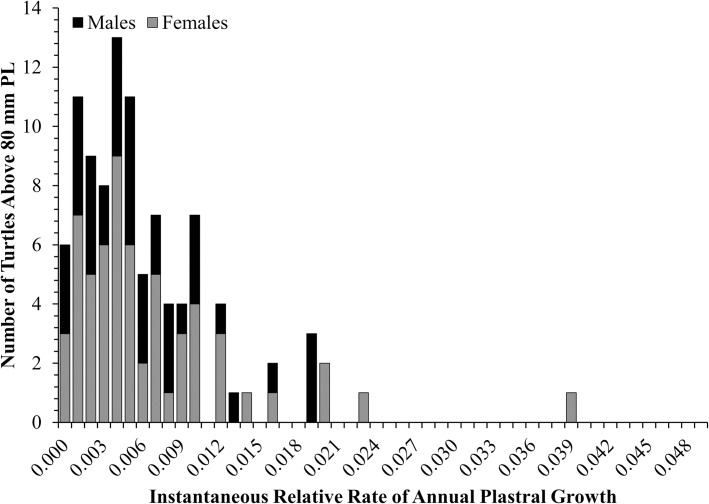
Histogram of Spotted Turtle (*Clemmys guttata*) instantaneous relative plastral growth rates (Δ*GR*). Δ*GR* was calculated from individuals with a plastron length > 80 mm and a capture interval greater than one year.

Nearly all (94 of 100) individuals showed some level of plastral growth after attaining maturity. However, most (58 of 100) had an instantaneous annual relative growth rate < 0.005, meaning the plastron length of over half of individuals increased by < 0.5% of their plastron length annually. Only 15 individuals increased by > 1% of their plastron length annually. Instantaneous relative growth rates were zero for only 6 individuals (3 males and 3 females) (Fig 3).

## Discussion

The most parsimonious model of growth included the effect of sex, showing males and females differ in mean growth trajectories. Litzgus and Brooks [5] studied an Ontario Spotted Turtle population and did not find a difference in growth between sexes. Such results hint at the potential advantages of accounting for sex in a single model, thus identifying subtle but important patterns and defining potential clinal or geographic variation. At the same time, we were unable to account for additional factors influencing turtle growth (e.g., seasonality, environmental conditions, etc.), so our model may overestimate the effect of sex. Considering environmental factors such as temperature, length of growing season, and food availability all affect turtle growth [53–55], the dissimilar growth patterns reported between the Ontario and Pennsylvania populations are likely a result of unknown clinal, seasonal, or environmental variation which should be the focus of future research.

Using growth curves from the interval model, we predicted females reach the minimum size of sexual maturity in ~ 7–12 yrs, which is in line with prior behavioral observations of reproduction in Spotted Turtles at the study site [33,34,49]. Interestingly, the interval model showed males maturing in ~ 11–34 yrs, the upper bounds of which may be unrealistic. One explanation is if males with the slowest growth suffer mortality before attaining maturity and drop out of the population, their slowed growth will result in a prediction of age at maturity which is never biologically realized. Additional data on the slowest growing males would be needed to refine estimates. At the same time, interval and age-specific growth curves provided statistically identical mean estimates of growth for females but not for males. Thus, the interval model simply may not have sufficient male data despite 23 yrs of capture-mark-recapture fieldwork. Considering estimating age from growth ring counts can be unreliable unless the number of growth rings are first calibrated to known-age individuals [56,57], and older turtles’ plastrons often become smooth and without visible rings due to wear [58], it is also possible the age-based model for males differs from the interval model due to annuli count error. When sufficient data is available, using a mixed-effects approach to better account for individual variation in growth models [e.g., 13,59] could lead to improved predictions.

After maturing, most Spotted Turtles continued growing slowly. Congdon et al. [11] assessed 13 populations of nine freshwater turtle species and found 19% of individuals on average ceased growing after maturity. In our study, only 6% of adult Spotted Turtles exhibited determinate growth in the strictest sense, although the growth rate was extremely low for most others (more than half of individuals increased < 0.5% of their plastron length annually). However, both our study and [11] may be biased towards indeterminate growth because small decreases in body size were set to zero [59].

Slow adult growth may be considered a type of determinate growth when small increases in size after maturity are established by environmental or genetic factors early on [60,61]. Extreme longevity in some turtle species coupled with seemingly trivial annual increases in growth can impact demography and fitness through an incremental attainment of larger size. The magnitude of the effects depends on the strength of the fecundity and survival advantage offered from increased body size [62], and the total increase in size achievable. In Common Snapping Turtles (*Chelydra serpentina*), slight growth following maturity resulted in increased survival and reproductive output [13]. Spotted Turtles at our study site have a weak but positive body-size clutch-size relationship [33], so there could be a slight fecundity advantage for the females that continue growing as adults. Also, older females continuing to grow as adults may have an advantage in egg size because larger female Spotted Turtles produce wider eggs [62].

For future turtle growth analyses, we recommend a two-step approach. First, the shape of the growth curve should be determined by comparing candidate models in an AIC framework [43,63,64]. Second, the most parsimonious model should be parameterized to account for sex or other factors potentially influencing growth patterns. In the present study, we incorporated sex, but depending on the research objectives, other factors could include population location, fall versus spring nest emergence, or year-cohort to account for annual environmental variation. The method would also be useful for modeling turtle growth in situations using headstarting, where there may be different growth patterns between captive-raised and wild turtles [65,66]. Integrated approaches to modeling growth incorporating skeletochronology and mixed effects to better account for individual variation are also well-suited [e.g., 20,59]. Future research on Spotted Turtles should examine how or if slow growth after maturity impacts population vital rates, thereby affecting demography through a fecundity or survival advantage offered to larger/older animals. It is also important to identify the environmental and genetic factors contributing to growth rate variation unaccounted for by sex.

## Supporting information

S1 Table Capture-mark-recapture data. Abbreviations are: Sex–F = Female, M = Male, Stage–A = Adult, J = Juvenile, DOCap = Date of Capture, DORec = Date of Recapture, PL1 and PL2 = Plastral Length in mm at DOCap and DORec, Age1 and Age2 = Estimated age at DOCap and DORec, INT = interval between captures in days, ΔPL = change in plastral length, ΔAge = change in age, ΔGR = instantaneous growth rate.
(DOCX)
