# Supplementary material for: Growing as slow as a turtle: Unexpected maturational differences in a small, long-lived species
Source: PLoS One. 2021 Nov 18;16(11):e0259978. doi: 10.1371/journal.pone.0259978 (PMC8601529; doi:10.1371/journal.pone.0259978)
Supplement: S1 Table — Abbreviations are: Sex–F = Female, M = Male, Stage–A = Adult, J = Juvenile, DOCap = Date of Capture, DORec = Date of Recapture, PL1 and PL2 = Plastral Length in mm at DOCap and DORec, Age1 and Age2 = Estimated age at DOCap and DORec, INT = interval between captures in days, ΔPL = change in plastral length, ΔAge = change in age, ΔGR = instantaneous growth rate. (DOCX) [file pone.0259978.s001.docx]

**S1 Table. Capture-mark-recapture data.**

Abbreviations are: Sex – F = Female, M = Male, Stage – A = Adult, J = Juvenile, DOCap = Date of Capture, DORec = Date of Recapture, PL1 and PL2 = Plastral Length in mm at DOCap and DORec, Age1 and Age2 = Estimated age at DOCap and DORec, INT = interval between captures in days, ΔPL = change in plastral length, ΔAge = change in age, ΔGR = instantaneous growth rate.

| **ID** | **Sex** | **Stage** | **DOCap** | **DORec** | **PL1** | **PL2** | **Age1** | **Age2** | **INT** | **ΔPL** | **ΔAge** | **ΔGR** |
| --- | --- | --- | --- | --- | --- | --- | --- | --- | --- | --- | --- | --- |
| 3 | F | A | 5/28/1965 | 6/17/1974 | 82 | 88.2 | 12 | 21 | 3307 | 6.2 | 9 | 0.00804 |
| 5 | F | A | 5/28/1965 | 3/29/1967 | 81 | 82.8 | 10 | 12 | 670 | 1.8 | 2 | 0.01197 |
| 6 | F | A | 5/17/1974 | 5/15/1980 | 95.5 | 97 | --- | --- | 2190 | 1.5 | --- | 0.00260 |
| 7 | F | J | 5/28/1965 | 4/26/1974 | 74.5 | 92.7 | 8 | 17 | 3255 | 18.2 | 9 | 0.02451 |
| 10 | F | A | 6/9/1965 | 3/23/1967 | 80 | 81 | 7 | 9 | 652 | 1 | 2 | 0.00695 |
| 12 | F | A | 6/25/1965 | 4/20/1985 | 92.1 | 98.3 | --- | --- | 7239 | 6.2 | --- | 0.00328 |
| 13 | F | A | 6/25/1965 | 3/11/1967 | 93.2 | 94.2 | 10 | 12 | 624 | 1 | 2 | 0.00624 |
| 17 | F | A | 4/16/1966 | 3/27/1985 | 88.4 | 93.9 | 14 | 33 | 6920 | 5.5 | 19 | 0.00318 |
| 18 | F | A | 4/23/1966 | 5/25/1967 | 92 | 92.1 | 12 | 13 | 397 | 0.1 | 1 | 0.00100 |
| 22 | F | A | 3/19/1966 | 4/25/1967 | 82.8 | 83 | 8 | 9 | 402 | 0.2 | 1 | 0.00219 |
| 23 | F | A | 3/19/1966 | 5/25/1967 | 88.8 | 89 | --- | --- | 432 | 0.2 | --- | 0.00190 |
| 24 | F | A | 3/19/1966 | 4/16/1985 | 89.8 | 97 | 12 | 31 | 6968 | 7.2 | 19 | 0.00404 |
| 26 | F | A | 3/19/1966 | 4/23/1966 | 90 | 90 | 14 | 14 | 35 | 0 | 0 | 0.00000 |
| 28 | F | A | 3/19/1966 | 3/23/1975 | 90.2 | 96.7 | 12 | 21 | 3291 | 6.5 | 9 | 0.00772 |
| 31 | F | A | 3/19/1966 | 6/6/1967 | 85.6 | 86.6 | 15 | 16 | 444 | 1 | 1 | 0.00955 |
| 32 | F | A | 3/19/1966 | 5/25/1967 | 81.9 | 82.7 | 11 | 12 | 432 | 0.8 | 1 | 0.00821 |
| 33 | F | J | 3/19/1966 | 4/20/1973 | 69.6 | 79.9 | 6 | 13 | 2589 | 10.3 | 7 | 0.01946 |
| 35 | F | A | 6/4/1966 | 3/30/1980 | 91.6 | 96.2 | --- | --- | 5048 | 4.6 | --- | 0.00354 |
| 37 | F | A | 6/11/1966 | 4/14/1983 | 93.1 | 98.9 | 16 | 33 | 6151 | 5.8 | 17 | 0.00359 |
| 39 | F | A | 6/11/1966 | 5/13/1967 | 85 | 85.1 | --- | --- | 336 | 0.1 | --- | 0.00128 |
| 43 | F | A | 8/1/1966 | 4/4/1980 | 88.2 | 93.5 | --- | --- | 4995 | 5.3 | --- | 0.00426 |
| 44 | F | A | 3/27/1967 | 5/29/1973 | 90.7 | 97.6 | 14 | 20 | 2255 | 6.9 | 6 | 0.01187 |
| 47 | F | J | 3/27/1967 | 5/21/1969 | 78.1 | 80 | 8 | 10 | 786 | 1.9 | 2 | 0.01116 |
| 49 | F | A | 3/27/1967 | 4/19/1985 | 90 | 95.8 | 12 | 30 | 6598 | 5.8 | 18 | 0.00345 |
| 50 | F | A | 6/2/1966 | 4/12/1974 | 80.9 | 90.2 | 10 | 18 | 2871 | 9.3 | 8 | 0.01383 |
| 52 | F | J | 3/29/1967 | 4/12/1974 | 78.8 | 88.6 | 9 | 16 | 2571 | 9.8 | 7 | 0.01664 |
| 55 | F | A | 6/21/1965 | 5/20/1969 | 83.6 | 88.9 | 10 | 14 | 1429 | 5.3 | 4 | 0.01570 |
| 58 | F | J | 3/29/1967 | 3/9/1973 | 74 | 85.3 | 7 | 13 | 2172 | 11.3 | 6 | 0.02388 |
| 59 | F | A | 6/29/1966 | 3/29/1967 | 94.4 | 94.4 | --- | --- | 273 | 0 | --- | 0.00000 |
| 61 | F | A | 6/10/1966 | 4/16/1981 | 86.5 | 91.5 | 12 | 29 | 5424 | 5 | 17 | 0.00378 |
| 65 | F | A | 3/30/1967 | 6/2/1967 | 84.5 | 85.2 | 10 | 10 | 64 | 0.7 | 0 | 0.04705 |
| 66 | F | A | 3/30/1967 | 4/16/1981 | 84.3 | 96.2 | 12 | 26 | 5131 | 11.9 | 14 | 0.00939 |
| 68 | F | A | 3/30/1967 | 5/13/1985 | 86.1 | 93 | 16 | 34 | 6619 | 6.9 | 18 | 0.00425 |
| 69 | F | A | 3/30/1967 | 5/2/1967 | 85.4 | 85.4 | 13 | 13 | 33 | 0 | 0 | 0.00000 |
| 71 | F | A | 3/11/1967 | 4/28/1967 | 85.4 | 85.5 | 14 | 14 | 48 | 0.1 | 0 | 0.00890 |
| 73 | F | J | 3/30/1967 | 6/16/1985 | 63.8 | 89.9 | 4 | 21 | 6653 | 26.1 | 17 | 0.01881 |
| 76 | F | J | 4/5/1967 | 6/14/1967 | 66.4 | 66.4 | 4 | 4 | 70 | 0 | 0 | 0.00000 |
| 77 | F | A | 4/5/1967 | 5/18/1967 | 83.5 | 83.5 | 9 | 9 | 43 | 0 | 0 | 0.00000 |
| 78 | F | J | 4/12/1967 | 5/7/1980 | 75.6 | 94.7 | 12 | 25 | 4774 | 19.1 | 13 | 0.01722 |
| 80 | F | A | 4/12/1967 | 3/12/1980 | 90.8 | 96 | 11 | 24 | 4718 | 5.2 | 13 | 0.00431 |
| 81 | F | A | 4/12/1967 | 4/1/1983 | 80.7 | 89.3 | 8 | 24 | 5833 | 8.6 | 16 | 0.00634 |
| 89 | F | A | 4/25/1967 | 6/2/1967 | 82.6 | 82.6 | 9 | 9 | 38 | 0 | 0 | 0.00000 |
| 90 | F | J | 4/25/1967 | 6/3/1975 | 66.5 | 83 | 4 | 12 | 2961 | 16.5 | 8 | 0.02732 |
| 92 | F | A | 4/28/1967 | 6/1/1967 | 90.7 | 90.7 | --- | --- | 34 | 0 | --- | 0.00000 |
| 112 | F | J | 6/1/1967 | 5/7/1986 | 53.7 | 97 | 4 | 23 | 6915 | 43.3 | 19 | 0.03121 |
| 116 | F | A | 6/6/1967 | 5/20/1968 | 83.3 | 85.7 | 10 | 11 | 349 | 2.4 | 1 | 0.02971 |
| 118 | F | A | 6/14/1967 | 4/10/1986 | 86.9 | 92.2 | 19 | 38 | 6875 | 5.3 | 19 | 0.00314 |
| 119 | F | A | 6/14/1967 | 5/2/1985 | 91.4 | 99.9 | 14 | 32 | 6532 | 8.5 | 18 | 0.00497 |
| 122 | F | J | 5/14/1968 | 5/21/1969 | 61.2 | 74.2 | 4 | 5 | 372 | 13 | 1 | 0.18899 |
| 124 | F | A | 5/21/1967 | 4/16/1981 | 88.9 | 96.7 | 12 | 23 | 5079 | 7.8 | 11 | 0.00604 |
| 125 | F | J | 8/23/1971 | 5/17/1973 | 62.3 | 71.8 | 3 | 5 | 633 | 9.5 | 2 | 0.08184 |
| 126 | F | A | 8/23/1971 | 5/3/1981 | 94.7 | 97.2 | 13 | 23 | 3541 | 2.5 | 10 | 0.00269 |
| 127 | F | A | 5/29/1972 | 4/11/1980 | 88.7 | 97.1 | 13 | 21 | 2874 | 8.4 | 8 | 0.01149 |
| 128 | F | A | 6/8/1972 | 4/20/1973 | 92.9 | 92.9 | 14 | 15 | 316 | 0 | 1 | 0.00000 |
| 137 | F | A | 5/10/1973 | 3/9/1979 | 85 | 95.1 | 9 | 15 | 2129 | 10.1 | 6 | 0.01925 |
| 138 | F | A | 5/12/1973 | 5/7/1980 | 94.2 | 95.9 | --- | --- | 2552 | 1.7 | --- | 0.00256 |
| 139 | F | A | 5/17/1973 | 4/5/1980 | 96.4 | 96.8 | 14 | 20 | 2515 | 0.4 | 6 | 0.00060 |
| 141 | F | A | 8/13/1969 | 4/25/1975 | 88.5 | 93.5 | --- | --- | 2081 | 5 | --- | 0.00964 |
| 142 | F | J | 8/13/1969 | 5/7/1985 | 77.9 | 94.1 | 7 | 23 | 5746 | 16.2 | 16 | 0.01200 |
| 144 | F | A | 5/21/1973 | 6/18/1975 | 93.6 | 93.7 | 14 | 16 | 758 | 0.1 | 2 | 0.00051 |
| 145 | F | A | 5/29/1973 | 5/20/1975 | 90.4 | 91.3 | 12 | 14 | 721 | 0.9 | 2 | 0.00502 |
| 146 | F | A | 5/31/1973 | 6/10/1986 | 97.5 | 98 | --- | --- | 4758 | 0.5 | --- | 0.00039 |
| 147 | F | A | 6/1/1973 | 4/26/1975 | 94.6 | 95.8 | 14 | 16 | 694 | 1.2 | 2 | 0.00663 |
| 151 | F | J | 6/15/1973 | 4/25/1975 | 78.6 | 79.3 | 7 | 9 | 679 | 0.7 | 2 | 0.00477 |
| 154 | F | A | 4/12/1974 | 6/4/1975 | 92.8 | 92.8 | --- | --- | 418 | 0 | --- | 0.00000 |
| 156 | F | A | 4/26/1974 | 5/2/1975 | 88.3 | 88.3 | 9 | 10 | 371 | 0 | 1 | 0.00000 |
| 157 | F | A | 4/26/1974 | 9/10/1987 | 93 | 96.5 | 10 | 23 | 4885 | 3.5 | 13 | 0.00276 |
| 161 | F | A | 5/17/1974 | 4/1/1983 | 95.3 | 98 | 13 | 22 | 3241 | 2.7 | 9 | 0.00315 |
| 166 | F | A | 5/21/1974 | 4/5/1980 | 96.2 | 96.8 | --- | --- | 2146 | 0.6 | 0 | 0.00106 |
| 167 | F | A | 5/21/1974 | 5/27/1975 | 98.7 | 99.7 | 13 | 14 | 371 | 1 | 1 | 0.00992 |
| 168 | F | A | 6/3/1974 | 5/27/1975 | 92.5 | 92.6 | 10 | 11 | 358 | 0.1 | 1 | 0.00110 |
| 172 | F | A | 6/3/1974 | 4/3/1976 | 84.4 | 87.9 | 8 | 10 | 670 | 3.5 | 2 | 0.02214 |
| 173 | F | A | 6/4/1974 | 4/19/1985 | 102.4 | 102.7 | --- | --- | 3972 | 0.3 | --- | 0.00027 |
| 177 | F | J | 3/23/1975 | 4/1/1983 | 51.7 | 93.4 | 4 | 12 | 2931 | 41.7 | 8 | 0.07365 |
| 180 | F | A | 4/25/1975 | 5/28/1975 | 99.1 | 99.1 | --- | --- | 33 | 0 | --- | 0.00000 |
| 184 | F | A | 5/22/1975 | 4/14/1979 | 99 | 100.7 | --- | --- | 1423 | 1.7 | --- | 0.00437 |
| 188 | F | A | 5/29/1975 | 4/11/1980 | 90.5 | 90.8 | --- | --- | 1779 | 0.3 | --- | 0.00068 |
| 194 | F | J | 5/26/1977 | 4/16/1981 | 74 | 96.9 | --- | --- | 1421 | 22.9 | --- | 0.06925 |
| 200 | F | J | 8/8/1979 | 4/5/1980 | 78.7 | 78.7 | 9 | 10 | 241 | 0 | 1 | 0.00000 |
| 201 | F | A | 4/14/1980 | 5/26/1981 | 96.8 | 101 | --- | --- | 407 | 4.2 | --- | 0.03809 |
| 202 | F | J | 4/4/1980 | 4/30/1984 | 65.1 | 79.8 | 9 | 13 | 1487 | 14.7 | 4 | 0.04998 |
| 207 | F | A | 5/3/1981 | 6/16/1981 | 95.5 | 96 | --- | --- | 44 | 0.5 | --- | 0.04332 |
| 211 | F | A | 3/26/1985 | 5/14/1985 | 97.5 | 97.5 | --- | --- | 49 | 0 | --- | 0.00000 |
| 220 | F | J | 5/3/1967 | 6/1/1988 | 57 | 96.9 | 4 | 25 | 7700 | 39.9 | 21 | 0.02515 |
| 222 | F | A | 5/26/1984 | 6/22/1985 | 83.4 | 83.4 | 8 | 9 | 392 | 0 | 1 | 0.00000 |
| 241 | F | A | 5/19/1975 | 6/16/1979 | 95 | 98.2 | --- | --- | 1489 | 3.2 | --- | 0.00812 |
| 250 | F | J | 5/15/1982 | 6/2/1985 | 73 | 75 | 6 | 9 | 1114 | 2 | 3 | 0.00886 |
| 258 | F | A | 5/16/1982 | 4/19/1986 | 94.6 | 95 | --- | --- | 1434 | 0.4 | --- | 0.00107 |
| 261 | F | A | 4/20/1985 | 4/29/1986 | 95.6 | 95.8 | --- | --- | 374 | 0.2 | --- | 0.00204 |
| 283 | F | A | 7/5/1985 | 4/10/1987 | 93.3 | 93.8 | --- | --- | 644 | 0.5 | --- | 0.00303 |
| 287 | F | A | 7/5/1974 | 4/13/1978 | 90.1 | 91.9 | --- | --- | 1378 | 1.8 | --- | 0.00524 |
| 288 | F | A | 7/5/1974 | 5/12/1976 | 97.7 | 98 | --- | --- | 677 | 0.3 | --- | 0.00165 |
| 300 | F | J | 4/26/1984 | 5/1/1986 | 58.3 | 70.2 | 5 | 7 | 735 | 11.9 | 2 | 0.09224 |
| 530 | F | A | 6/8/1972 | 4/26/1975 | 80 | 84.7 | 7 | 10 | 1052 | 4.7 | 3 | 0.01981 |
| 548 | F | A | 5/25/1974 | 5/21/1985 | 92.4 | 94 | 12 | 23 | 4014 | 1.6 | 11 | 0.00156 |
| 589 | F | A | 3/25/1975 | 5/7/1980 | 89.9 | 90.3 | --- | --- | 1870 | 0.4 | --- | 0.00087 |
| 1 | M | A | 5/28/1965 | 5/23/1975 | 82.8 | 86 | 10 | 20 | 3647 | 3.2 | 10 | 0.00380 |
| 2 | M | A | 5/28/1965 | 3/29/1967 | 86.5 | 89.5 | 11 | 13 | 670 | 3 | 2 | 0.01857 |
| 8 | M | A | 4/23/1966 | 4/28/1967 | 86.2 | 86.8 | --- | --- | 370 | 0.6 | --- | 0.00684 |
| 9 | M | A | 4/20/1973 | 3/25/1975 | 83.2 | 83.3 | --- | --- | 704 | 0.1 | --- | 0.00062 |
| 11 | M | A | 6/9/1965 | 3/20/1974 | 84 | 91 | 10 | 19 | 3206 | 7 | 9 | 0.00911 |
| 14 | M | A | 4/22/1966 | 3/29/1967 | 82.1 | 82.9 | 12 | 13 | 341 | 0.8 | 1 | 0.01038 |
| 15 | M | A | 4/16/1966 | 6/5/1967 | 86.4 | 86.4 | 15 | 16 | 415 | 0 | 1 | 0.00000 |
| 16 | M | A | 4/16/1966 | 6/1/1973 | 85.8 | 90.3 | 6 | 12 | 2603 | 4.5 | 6 | 0.00717 |
| 19 | M | J | 3/19/1966 | 6/2/1972 | 79 | 84.3 | 7 | 13 | 2267 | 5.3 | 6 | 0.01045 |
| 20 | M | A | 3/19/1966 | 5/3/1967 | 89 | 89.2 | 11 | 12 | 410 | 0.2 | 1 | 0.00200 |
| 21 | M | A | 3/19/1966 | 4/5/1980 | 83 | 90.7 | 8 | 22 | 5131 | 7.7 | 14 | 0.00631 |
| 27 | M | A | 3/19/1966 | 6/16/1975 | 80 | 83.8 | 10 | 19 | 3376 | 3.8 | 9 | 0.00502 |
| 29 | M | A | 3/19/1966 | 4/12/1975 | 82.9 | 89.8 | 11 | 20 | 3311 | 6.9 | 9 | 0.00881 |
| 30 | M | A | 3/19/1966 | 5/26/1967 | 84.2 | 84.7 | 13 | 14 | 433 | 0.5 | 1 | 0.00499 |
| 34 | M | A | 3/19/1966 | 6/4/1966 | 81.8 | 82 | 10 | 10 | 77 | 0.2 | 0 | 0.01158 |
| 42 | M | A | 8/1/1966 | 6/21/1967 | 81.7 | 81.9 | 10 | 11 | 324 | 0.2 | 1 | 0.00275 |
| 45 | M | A | 3/27/1967 | 4/5/1985 | 81.5 | 87.2 | 13 | 31 | 6584 | 5.7 | 18 | 0.00375 |
| 46 | M | A | 3/27/1967 | 5/8/1985 | 83.2 | 89.2 | 14 | 32 | 6617 | 6 | 18 | 0.00384 |
| 48 | M | A | 3/27/1967 | 6/28/1967 | 80.2 | 80.5 | 8 | 8 | 93 | 0.3 | 0 | 0.01465 |
| 53 | M | A | 3/29/1967 | 3/13/1985 | 87.2 | 94.8 | 10 | 28 | 6559 | 7.6 | 18 | 0.00465 |
| 54 | M | A | 3/29/1967 | 5/4/1973 | 84.1 | 89 | 9 | 15 | 2228 | 4.9 | 6 | 0.00928 |
| 56 | M | A | 3/29/1967 | 6/21/1967 | 87.8 | 87.8 | 13 | 13 | 84 | 0 | 0 | 0.00000 |
| 57 | M | J | 3/29/1967 | 5/29/1972 | 78.7 | 82.8 | 9 | 14 | 1888 | 4.1 | 5 | 0.00982 |
| 60 | M | A | 3/29/1967 | 6/6/1967 | 81.8 | 81.8 | 7 | 7 | 69 | 0 | 0 | 0.00000 |
| 62 | M | J | 3/30/1967 | 5/25/1967 | 76.8 | 76.8 | 6 | 6 | 56 | 0 | 0 | 0.00000 |
| 63 | M | A | 3/30/1967 | 6/16/1972 | 87.4 | 94.8 | --- | --- | 1905 | 7.4 | --- | 0.01557 |
| 64 | M | A | 3/30/1967 | 5/18/1967 | 80.2 | 80.2 | 10 | 10 | 49 | 0 | 0 | 0.00000 |
| 67 | M | A | 3/30/1967 | 6/21/1967 | 85.4 | 85.4 | 14 | 14 | 83 | 0 | 0 | 0.00000 |
| 70 | M | A | 6/4/1966 | 6/9/1972 | 80.8 | 85.5 | 12 | 18 | 2197 | 4.7 | 6 | 0.00939 |
| 72 | M | A | 3/11/1967 | 4/20/1974 | 81.8 | 93.2 | 13 | 20 | 2597 | 11.4 | 7 | 0.01834 |
| 75 | M | A | 3/30/1967 | 5/3/1981 | 80 | 86 | 7 | 21 | 5148 | 6 | 14 | 0.00513 |
| 82 | M | J | 4/12/1967 | 5/11/1974 | 73.8 | 78.4 | 8 | 16 | 2586 | 4.6 | 8 | 0.00853 |
| 83 | M | A | 4/12/1967 | 6/12/1967 | 88.3 | 88.3 | 12 | 12 | 61 | 0 | 0 | 0.00000 |
| 86 | M | A | 4/15/1967 | 4/20/1984 | 81 | 92 | 8 | 25 | 6215 | 11 | 17 | 0.00748 |
| 88 | M | A | 4/25/1967 | 5/25/1967 | 86.5 | 86.5 | 11 | 11 | 30 | 0 | 0 | 0.00000 |
| 93 | M | A | 4/28/1967 | 5/29/1972 | 82.4 | 87.8 | 9 | 14 | 1858 | 5.4 | 5 | 0.01247 |
| 96 | M | J | 4/28/1967 | 3/10/1973 | 74.1 | 85.6 | 7 | 13 | 2143 | 11.5 | 6 | 0.02457 |
| 104 | M | A | 5/10/1967 | 3/14/1986 | 82.6 | 90 | --- | --- | 6883 | 7.4 | --- | 0.00455 |
| 107 | M | A | 5/13/1967 | 4/16/1981 | 81.9 | 90.8 | 8 | 22 | 5087 | 8.9 | 14 | 0.00740 |
| 114 | M | J | 6/1/1967 | 5/20/1968 | 79 | 81 | 8 | 9 | 354 | 2 | 1 | 0.02578 |
| 123 | M | A | 5/21/1969 | 3/14/1986 | 83.7 | 102 | 8 | 25 | 6141 | 18.3 | 17 | 0.01175 |
| 129 | M | A | 6/9/1972 | 3/17/1979 | 87.9 | 90.5 | 10 | 17 | 2472 | 2.6 | 7 | 0.00430 |
| 132 | M | A | 3/9/1973 | 4/4/1980 | 86.8 | 89.5 | 9 | 16 | 2583 | 2.7 | 7 | 0.00433 |
| 134 | M | A | 4/20/1973 | 4/19/1985 | 87.3 | 89.2 | 9 | 21 | 4382 | 1.9 | 12 | 0.00179 |
| 135 | M | A | 5/4/1973 | 3/29/1980 | 90.5 | 90.7 | 10 | 17 | 2521 | 0.2 | 7 | 0.00032 |
| 153 | M | A | 3/20/1974 | 4/17/1985 | 100.5 | 100.7 | --- | --- | 4046 | 0.2 | --- | 0.00018 |
| 159 | M | A | 5/16/1974 | 4/15/1985 | 92.1 | 93 | --- | --- | 3987 | 0.9 | --- | 0.00089 |
| 170 | M | J | 6/3/1974 | 3/31/1986 | 71.3 | 94.1 | 4 | 16 | 4319 | 22.8 | 12 | 0.02345 |
| 171 | M | A | 6/3/1974 | 4/23/1985 | 88.4 | 94 | --- | --- | 3977 | 5.6 | --- | 0.00564 |
| 175 | M | A | 6/5/1974 | 4/1/1983 | 92 | 95.3 | --- | --- | 3222 | 3.3 | --- | 0.00399 |
| 176 | M | J | 3/22/1975 | 4/25/1975 | 78.4 | 78.4 | --- | --- | 34 | 0 | --- | 0.00000 |
| 181 | M | A | 5/2/1975 | 4/20/1979 | 91 | 91.5 | --- | --- | 1449 | 0.5 | --- | 0.00138 |
| 193 | M | J | 5/26/1977 | 5/2/1985 | 75 | 83.6 | 12 | 20 | 2898 | 8.6 | 8 | 0.01367 |
| 196 | M | A | 3/9/1974 | 5/14/1985 | 90.6 | 90.6 | --- | --- | 4084 | 0 | --- | 0.00000 |
| 205 | M | A | 5/26/1984 | 4/23/1985 | 92 | 92 | --- | --- | 332 | 0 | --- | 0.00000 |
| 208 | M | A | 4/20/1984 | 4/19/1986 | 89.5 | 90 | 10 | 12 | 729 | 0.5 | 2 | 0.00279 |
| 209 | M | A | 5/26/1984 | 4/15/1985 | 87.1 | 87.4 | 10 | 11 | 324 | 0.3 | 1 | 0.00387 |
| 234 | M | A | 5/9/1970 | 8/10/1974 | 88 | 95.2 | 14 | 18 | 1554 | 7.2 | 4 | 0.01847 |
| 242 | M | A | 6/3/1974 | 3/25/1975 | 87 | 87.6 | --- | --- | 295 | 0.6 | --- | 0.00850 |
| 244 | M | A | 5/14/1982 | 4/26/1986 | 83.2 | 83.2 | --- | --- | 1443 | 0 | --- | 0.00000 |
| 280 | M | A | 7/4/1985 | 4/26/1986 | 89 | 89 | --- | --- | 296 | 0 | --- | 0.00000 |
| 291 | M | A | 9/11/1974 | 4/26/1986 | 88.1 | 90.9 | --- | --- | 4245 | 2.8 | --- | 0.00269 |
| 293 | M | A | 5/9/1969 | 3/13/1970 | 82.5 | 83 | --- | --- | 308 | 0.5 | --- | 0.00716 |
| 296 | M | A | 6/3/1974 | 3/25/1975 | 87 | 87 | --- | --- | 295 | 0 | --- | 0.00000 |
| 575 | M | A | 5/25/1975 | 3/14/1979 | 85.6 | 86 | --- | --- | 1389 | 0.4 | --- | 0.00123 |
